# Magnesium lithospermate B improves renal hemodynamics and reduces renal oxygen consumption in 5/6th renal ablation/infarction rats

**DOI:** 10.1186/s12882-019-1221-5

**Published:** 2019-02-12

**Authors:** Pinglan Lin, Ming Wu, Junyan Qin, Jing Yang, Chaoyang Ye, Chen Wang

**Affiliations:** 1 Department of Nephrology, Shuguang Hospital Affiliated to Shanghai University of Traditional Chinese Medicine, No.528 Zhangheng Road, Pudong District, Shanghai, 201203 People’s Republic of China; 2 TCM Institute of Kidney Disease, Shanghai University of Traditional Chinese Medicine, Shanghai, People’s Republic of China; 3 Key Laboratory of Liver and Kidney Diseases (Shanghai University of Traditional Chinese Medicine) Ministry of Education, Shanghai, People’s Republic of China; 4 Shanghai Key Laboratory of Traditional Chinese Clinical Medicine, Shuguang Hospital Affiliated to Shanghai University of Traditional Chinese Medicine, Shanghai, People’s Republic of China

**Keywords:** Magnesium lithospermate B, Renal blood flow, Renal oxygen consumption, Hypoxia, CRF

## Abstract

**Background:**

Magnesium lithospermate B (MLB) can promote renal microcirculation. The aim of the current project was to study whether MLB improves renal hemodynamics, oxygen consumption and subsequently attenuates hypoxia in rats induced by 5/6th renal Ablation/Infarction(A/I).

**Methods:**

Chronic renal failure (CRF) was induced in male SD rats by the 5/6 (A/I) surgery. 30 rats were randomly divided into three groups: sham group, 5/6 (A/I) + vehicle group (CRF group) and 5/6 (A/I) + MLB (CRF + MLB) group. 28 days after the surgery, rats were given with saline or 100 mg/kg MLB by i.p. injection for 8 weeks. The 24-h urinary protein (24hUp), serum creatinine (Scr), blood urine nitrogen (BUN), systolic blood pressure (SBP) and diastolic blood pressure (DBP) were measured. The protein expression of Fibronectin (FN), Collagen-I (Col-I), Connective Tissue Growth Factor(CTGF) and Interleukin-6 (IL-6) were measured by Western blot. Renal blood flow (RBF) and renal O_2_ consumption (QO_2_) indicated as sodium reabsorption (QO_2_/TNa) were detected before sacrifice. Renal hypoxia was assessed by measuring the protein expression of nNOS, HIF-1α and VEGF.

**Results:**

MLB significantly reduced 24hUp, Scr, BUN, SBP and DBP levels in rats with CRF. The expression of FN, Col-I, CTGF and IL-6 were down-regulated by MLB treatment in rats with CRF. In comparison to sham operated rats, 5/6 (A/I) rats had significantly lower RBF, and MLB significantly increased RBF in rats with CRF. Moreover, QO_2_/TNa was higher in the CRF group as compared to that in the sham group, and it was significantly attenuated in the CRF + MLB group. MLB reversed the expression of nNOS (neuronal nitric oxide synthase), HIF-1α (hypoxia inducible factor-1) and VEGF in rats with CRF.

**Conclusions:**

MLB improves renal function, fibrosis and inflammation in CRF rats induced by 5/6 (A/I), which is probably related to the increase in RBF, reduction of oxygen consumption and attenuation of renal hypoxia in the remnant kidney with CRF.

## Background

Chronic kidney disease (CKD) is defined as the glomerular filtration rate (GFR) < 60 mL/min per 1·73 m^2^ or signs of renal damage, or both, for at least 3 months duration [1]. CKD causes a substantial economic burden, and according to the estimation of WHO the death rate associated with CKD will increase to 14 per 100,000 people in 2030 [1, 2]. Renal fibrosis and inflammation, representing the unsuccessful wound-healing of kidney tissues, are the final common pathological features of different types of chronic kidney disease [1].

Kidney is a hypoxia-sensitive organ, and hypoxia in the kidney can be caused by inadequacy of oxygen supply and increased consumption of oxygen [3, 4]. Based on the Brenner’s hyperfiltration theory, the tubulointerstitial damages in CKD impairs blood flow in the peritubular capillary and induces ischemic injuries which leads to renal function decline and loss of nephrons [3, 5].

Magnesium lithospermate B (MLB) is a nature product from the aqueous extracts of traditional Chinese herbal medicine, *Salviae miltiorrhizae* Bge. [6]. MLB displayed renal protective effects in several rodent models of kidney disease. In CKD rats induced by 5/6thnephrectomy, MLB improved renal function as shown by decreased serum creatinine (Scr) and blood urea nitrogen (BUN) levels, which was correlated with reduced mesangial proliferation, tubulointerstitial lesions and glomerular sclerotic lesions [7, 8]. In streptozotocin-induced diabetic rats, MLB also displayed a reno-protective property as shown by reduced microalbuminuria, glomerular hypertrophy, and mesangial expansion. These beneficial effects of MLB in diabetic kidneys was associated with decreased expression of renal malondialdehyde (MDA), TGF-β1, fibronectin, and collagen [9]. It was shown in another report that after 45 min treatment with MLB, renal cortical microperfusion was significantly increased in healthy rat kidneys [10]. Moreover, MLB increased renal blood flow in adenine induced CKD rats [11]. However, it is not known whether MLB improves renal hypoxia through ameliorating renal microcirculatory system in diseased kidneys.

In the current study, we aimed to study whether MLB improves renal hypoxia and protects renal function in 5/6 ablation/infarction (A/I) rats through ameliorating renal hemodynamics and attenuating renal oxygen consumption.

## Methods

### Animals

Male Sprague-Dawley (SD) rats (SPF grade) weighted between 190 and 210 g were purchased from Shanghai SLAC Laboratory Animal Co., Ltd. Animals were housed in the animal center of Shanghai University of Traditional Chinese Medicine according to local regulations and guidelines.

### 5/6^th^Renal ablation and infarction (a/I) surgery

Rats were anesthetized by sodium pentobarbital (20 mg/kg, i.p.), and placed on the temperature controlled surgical table. The renal artery of left kidney was exposed after a small flank incision. Two branches of the left renal artery were ligated with 4–0 silk sutures. The left kidney was then gently returned to the body and the incision was closed. After one week, a right flank incision was made, and the adrenal gland was separated from the right kidney. The right kidney was removed after the right renal pedicle was ligated. Control rats underwent the same anesthetic procedures and sham operation were performed on both side of kidneys. The rats were kept warm in an incubator until fully ambulatory.

### The animal study protocol

30 rats were randomly divided into three groups: (I) sham group (*n* = 10), (II) 5/6 (A/I) + vehicle group (CRF group), and (III) 5/6 (A/I) + MLB (CRF + MLB) group. 28 days after the surgery, rats were treated with saline or 100 mg/kg MLB daily by intraperitoneally (i.p.) injection for 8 weeks. 24-h urine samples were collected one day before sacrifice. 0.8%Sodium pentobarbital was used as anesthesia before sacrificed by intraperitoneally (i.p.) injection. Blood samples and kidney tissues were collected after opening the abdominal cavity.

Animal experiments described herein were endorsed by the animal experimentation ethics committee of Shanghai University of Traditional Chinese Medicine.

### Renal function and O_2_ consumption measurement

Serum creatinine (Scr), blood urea nitrogen (BUN) and 24-h urinary protein (24hUp) were detected by Automatic biochemical analyzer (AU680, Beckman Coulter).

Rats were anesthetized with sodium pentobarbital (20 mg/kg, i.p.) before the O_2_ consumption measurement. The left renal blood flow (RBF, ml/min) was monitored with a perivascular ultrasonic transit-time flow probe (Transonics T420, Ithaca, USA) which was connected to a computer for continuous recording. Proximal left renal vein was used for sampling of venous blood. Blood samples were taken from the femoral artery and renal vein for measurements of total arterial blood hemoglobin (tHb), (O_2_Hb), (pO_2_), (pCO_2_), pH, [Na^+^], [K^+^], [HCO_3_^−^] with the blood gas analyzer and biochemical multiple test cards (i-STAT EG7, U.S.A, Abbott). O_2_ content (O_2_ct) was calculated by the formula:O2ct (ml/ml blood) = (1.39 xtHbxO_2_Hb% + pO_2_x0.03)/100. The total left kidney O_2_ consumption (QO_2_, ml/min) was calculated from A-V difference in O_2_ content multiplied by RBF. TNa is equal to the total amount of sodium filtered (FNa) minus the amount of sodium excreted in the urine (UNaV). Systolic blood pressure was measured by the tail-cuff method. Cuff inflation/deflation rates and maximum cuff pressure were controlled by a programmed electro-sphygmomanometer Softron BP-2010A (Softron Biotechnology, Beijing, China). The calculated value is the mean of 3 to 5 recordings performed at the same time during seven days before sacrificed.

### Masson’s trichrome and Immunohistochemical staining

Kidneys were fixed in 4% paraformaldehyde and embedded in paraffin. Four-μm-thick sections of paraffin-embedded kidney tissue were subjected to immunohistochemical staining with anti-VEGF (1:1000, A0280, Abclonal) antibodies. Masson’s trichrome staining was performed using a standard protocol as described by Livingston et al. [12]. Briefly, the tissue was stained with hematoxylin, and then with ponceau red liquid dye acid complex, which was followed by incubation with phosphomolybdic acid solution. Finally, the tissue was stained with aniline blue liquid and acetic acid. Images were obtained with the use of a microscope (Nikon 80i, Tokyo, Japan).

### Western blotting analysis

Renal protein was extracted from the medulla and cortex of rat kidneys. The protein concentration was measured by the Bradford method, and the supernatant was denatured at 95 °C for 5 min in Laemmli sample buffer. Samples were subjected to SDS-PAGE gels. After electrophoresis, proteins were electro-transferred to a polyvinylidene difluoride membrane (Merck), which was incubated in the blocking buffer (5% non-fat milk, 20 mM Tris-HCl, 150mMNaCl, PH = 8.0, 0.01%Tween 20) for 1 h at room temperature and was followed by incubation with anti-fibronectin (1:1000, ab23750, Abcam) or anti-Collagen-I (1:1000, sc-293,182, Santa Cruz) or anti-CTGF (1:1000, sc-373,936, Santa Cruz) anti-nNOS (1:1000, 4236 s, CST) or anti-HIF-1α (1:1000, ab2185, Abcam) or anti-VEGF (1:1000,A0280, Abclonal) overnight at 4 °C. Binding of the primary antibody was detected by an enhanced chemiluminescence method (BeyoECL Star, P0018A, Byotime) using horseradish peroxidase-conjugated secondary antibodies (goat anti-rabbit IgG,1:1000, Proteintech). The quantification of protein expression was performed using Quantity One Analyzer (Bio-Rad).

### Statistical analysis

Results were presented as mean ± SE. Differences among multiple groups were analyzed by one-way analysis of variance (ANOVA) and comparison between two groups was performed by paired Student t-test or unpaired student t-test. Using statistic software SPSS 18.0 (SPSS Inc., Chicago, IL). A *P* value of lower than 0.05 was considered statistically significant.

## Results

The renal ablation/infarction (A/I) model of chronic kidney disease (CKD) was established in male SD rats weighted 190–210 g, which were randomly divided into three groups: (I) sham operation, (II) 5/6 renal ablation/infarction (A/I) operation, (III) 5/6 A/I operation +Magnesium Lithospermate B (MLB). 28 days after the operation, rats were treated with vehicle or 100 mg/kg MLB by i.p. once daily for 8 weeks.

### Renal function decline was retarded in the CRF rats with MLB treatment

**Fig. 1:**
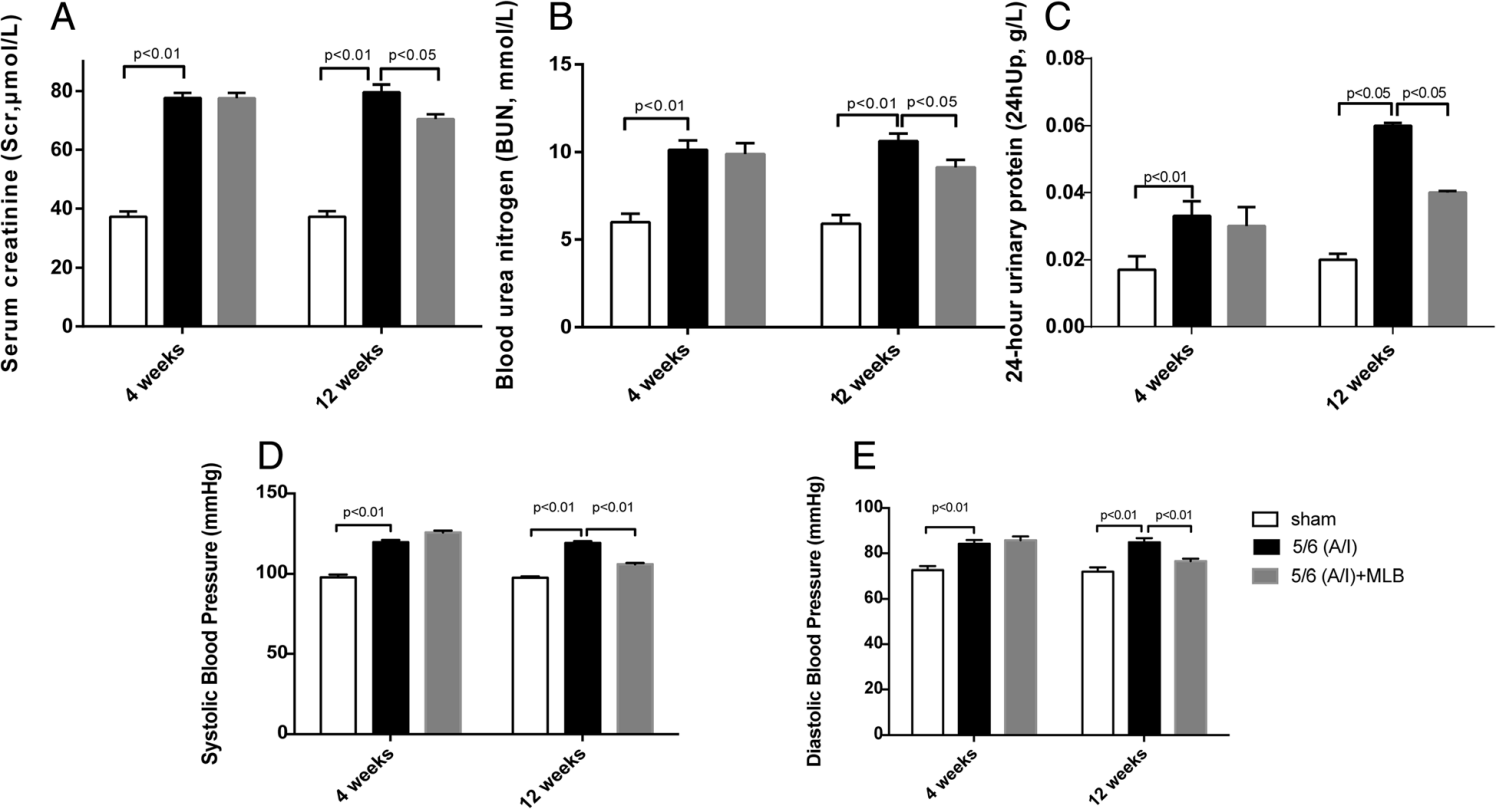
Renal function in rats with sham or renal ablation/infarction (A/I) operation. 4 weeks after sham or 5/6 ablation/infarction (A/I) operation, SD rats were treated with vehicle or 100 mg/kg MLB by i.p. once daily for another 8 weeks. The levels of serum creatinine (Scr; **a**),Blood urea nitrogen (BUN; **b**),24-h urinary protein (24hUp; **c**), Systolic Blood Pressure (SBP; **d**) and Diastolic Blood Pressure (DBP; **e**) were measured at 4 weeks and 12 weeks after operation. Data represent mean ± SE

Figure 1a shows that the serum creatinine (Scr) levels in the 5/6 (A/I) model group was significantly (*p* < 0.01) higher than that in sham group at 4 weeks after the surgery. 8 weeks of treatment with MLB significantly(*p* < 0.05) reduced the serum creatinine (Scr) levels by 9.19% in CRF rats at 12 weeks after the surgery.

Rats with 5/6 (A/I) operation had significantly higher levels of Blood urea nitrogen (BUN) than rats in the sham group at 4 weeks after the surgery (Fig. 1). The BUN levels were 14.11% (p < 0.05) lower in the 5/6 (A/I) + MLB group than in the 5/6 (A/I) model group after 8-weeks treatment with MLB (Fig. 1b).

As showed in Fig. 1c, the 5/6(A/I) operation significantly increased the 24-h urine protein excretion as compared with the sham operation in rats at 4 weeks and 12 weeks after operation. The 24-h urine protein excretion in the 5/6 (A/I) + MLB group was 30% lower than that in the 5/6 (A/I) model group after 8-weeks intervention with MLB.

SBP and DBP were significantly increased in rats at 4 weeks and 12 weeks after the 5/6 (A/I) operation(Fig. 1d and e).With MLB treatment, SBP and DBP were reduced by 11.16% (*p* < 0.05) and 9.8% (*p* < 0.05) respectively in CRF rats at 12 weeks after the operation.

### MLB treatment attenuated renal fibrosis and inflammation in the CRF rats

To further determine the effect of MLB in the CRF rats, we measured the collagen deposition by Masson’s trichrome staining and the expression of several markers for renal fibrosis and inflammation.

**Fig. 2:**
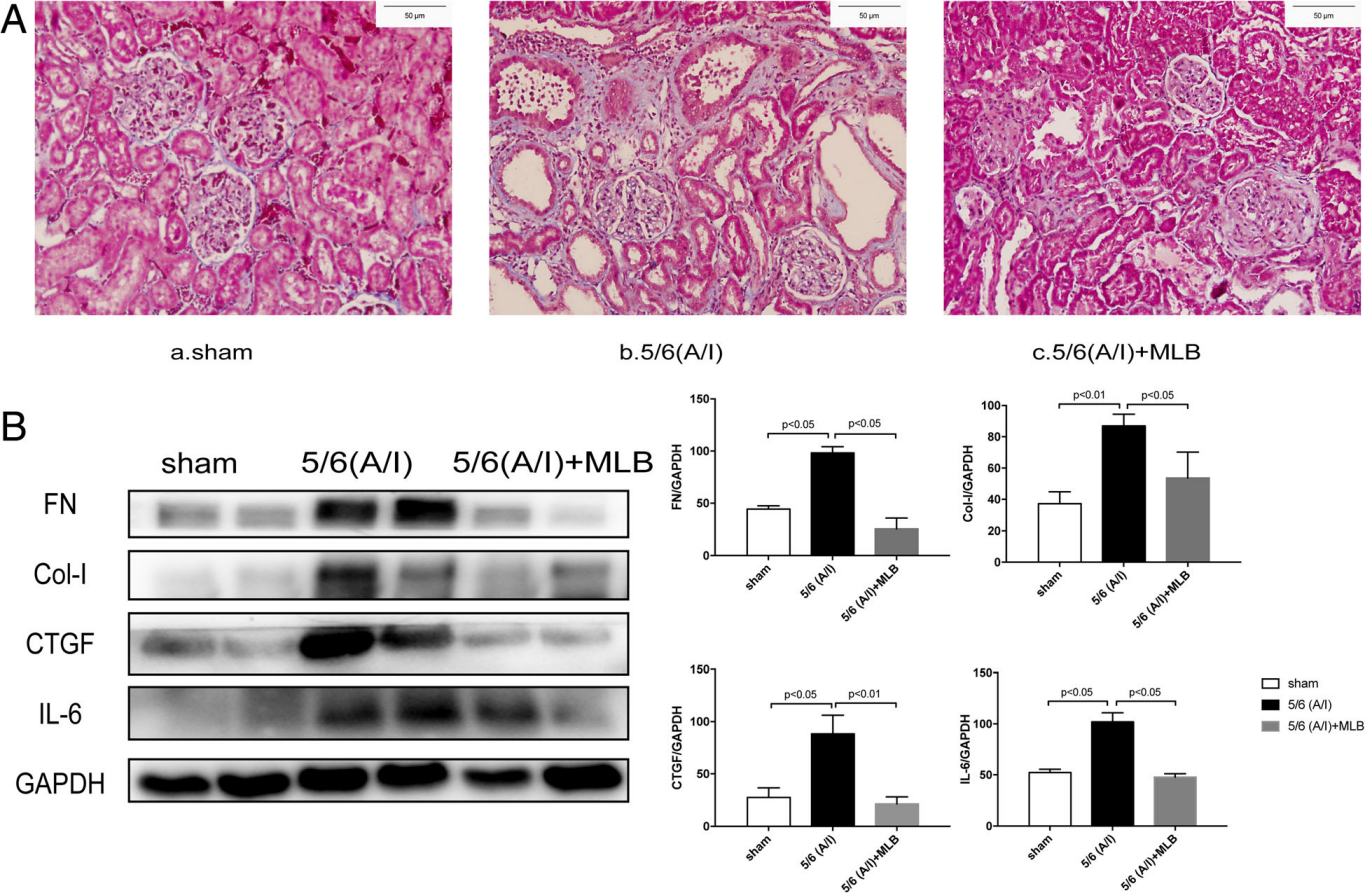
Renal fibrosis and inflammation in rats with sham or renal ablation/infarction (A/I) operation. 4 weeks after sham or 5/6 ablation/infarction (A/I) operation, SD rats were treated with vehicle or 100 mg/kg MLB by i.p. once daily for another 8 weeks. Representative images of Masson’s trichrome staining (**a**). The expression of FN, Col-I, CTGF and IL-6 in renal tissues were analyzed by Western blotting (**b**). One representative of three independent experiments is shown. The result of Western blotting was quantified by densitometry. Data represent mean ± SE

As shown by Fig. 2a, there was an increase in blue Masson’s trichrome staining on the kidney of CRF rats as compared with that of sham rats at 12 weeks after the operation. After 8-weeks MLB treatment, Masson’s trichrome staining was reduced in the renal interstitial area of CRF rats. In parallel, the protein expression of FN, Col-I, and CTGF was significantly increased in CFR rat kidneys as compared with that in the sham-operated rat kidneys, and the expression of these fibrotic markers in CRF rat kidneys were significantly reduced by the MLB treatment at 12 weeks after the operation (Fig. 2b).

Moreover, the expression of IL-6,an inflammation marker, was up-regulated in the 5/6 (A/I) model group as compared with that in the sham group (*p* < 0.05). The treatment with MLB for 8-weeks significantly down-regulated the expression of IL-6 in the rat kidney with 5/6 (A/I) operation (Fig. 2b).

### Deterioration of the renal blood flow and **r**emnant renal oxygen consumption (QO_2_ /T_Na_) was improved in the CRF rats with MLB treatment

**Fig. 3:**
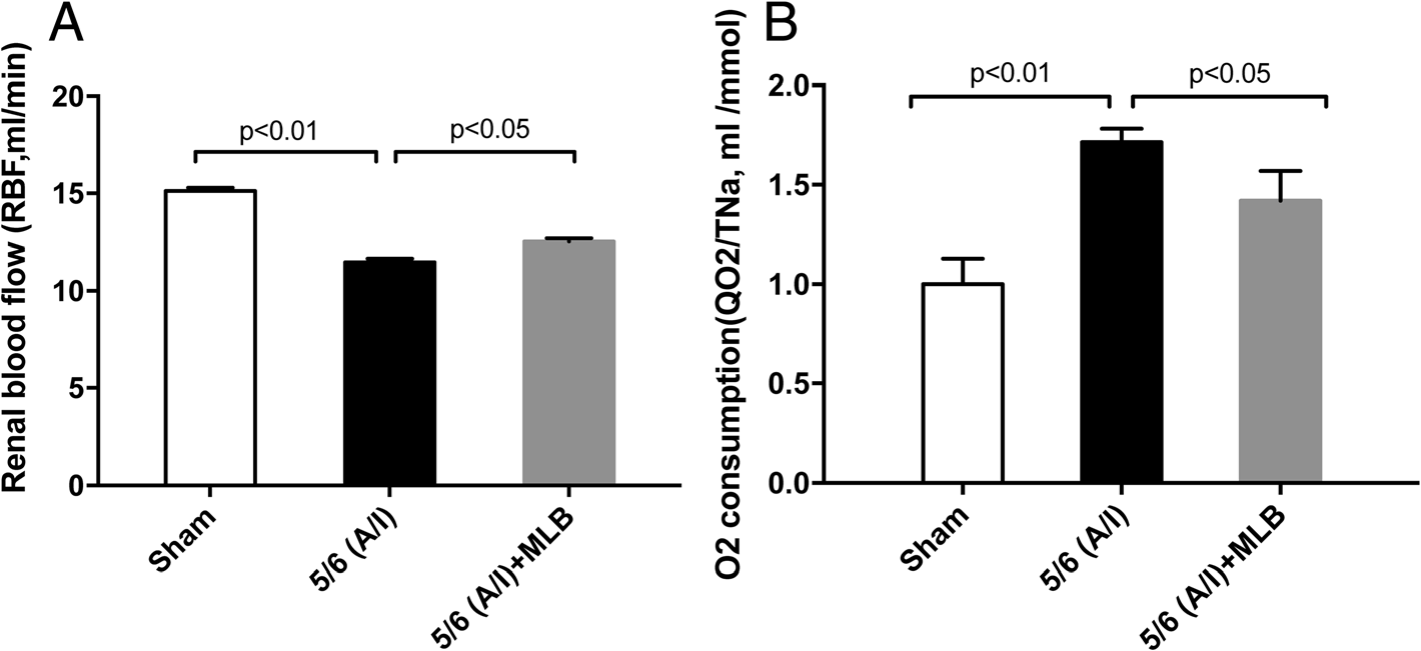
Renal blood flow (RBF) and renal O_2_ consumption in rats with sham or renal ablation/infarction (A/I) operation.4 weeks after sham or 5/6 ablation/infarction (A/I) operation, SD rats were treated with vehicle or 100 mg/kg MLB by i.p. once daily for another 8 weeks. Renal blood flow (RBF; **a**)and renal O_2_ consumption (QO_2_; **b**) indicated as sodium reabsorption (QO_2_/TNa) were detected before sacrifice. Data represent mean ± SE

After MLB treatment, we measured renal blood flow (RBF) and oxygen consumption which was reflected by sodium transport efficiency (QO_2_/T_Na_). The rate of RBF is significantly lower in the 5/6 (A/I) model group as compared to that in the sham group(11.48 ± 0.55 vs. 15.13 ± 0.49, ml/min, *p* < 0.01) (Fig. 3a). The rate of RBF was increased to 12.52 ± 0.52 ml/min (*p* < 0.05) in the 5/6 (A/I) + MLB group as compared to that in the5/6 (A/I) model group (Fig. 3a). The 5/6 (A/I) operation induced the value of QO_2_/T_Na_ in rat kidneys than that in the sham group (1.00 ± 0.13 vs. 1.72 ± 0.20, ml/mmol, *p* < 0.01) (Fig. 2b). QO_2_/T_Na_ was significantly lower in the 5/6 (A/I) + MLB group than that in the 5/6 (A/I) model group (1.42 ± 0.15 vs. 1.72 ± 0.20, ml/mmol, *p* < 0.05)after 8 weeks treatment with MLB or vehicle (Fig. 3b).

### Effects of MLB on the protein expression of nNOS, HIF-1α and VEGF in the remnant kidneys

**Fig. 4:**
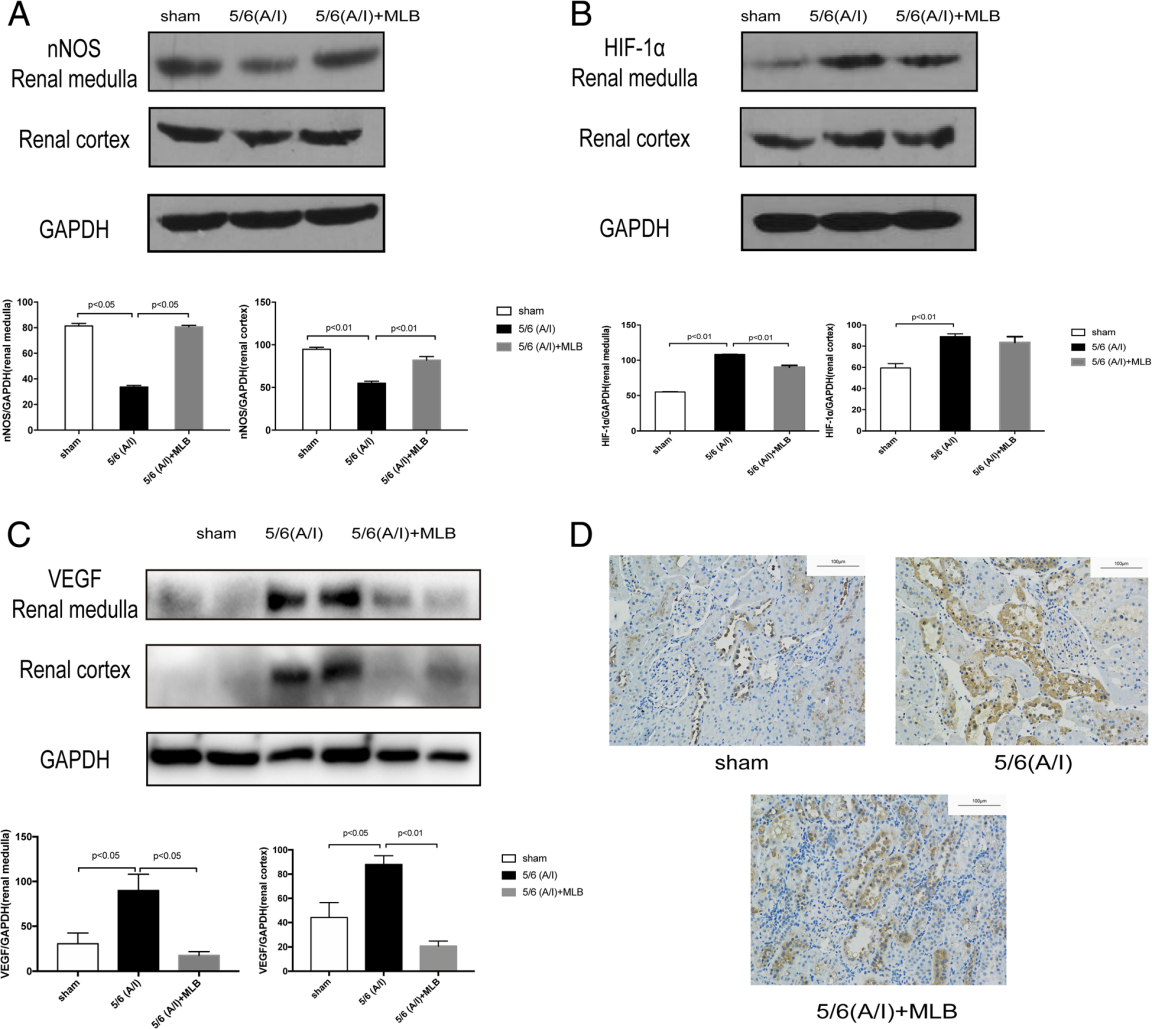
The expression of nNOS,HIF-1α and VEGF in rats with sham or renal ablation/infarction (A/I) operation.4 weeks after sham or 5/6 ablation/infarction (A/I) operation, SD rats were treated with vehicle or 100 mg/kg MLB by i.p. once daily for another 8 weeks. Kidney tissues were collected after sacrifice and protein analysis was performed by Western blotting. The expression of nNOS (**a**), HIF-1α (**b**) and VEGF (**c**) in renal medulla and cortex. Representative images of immunohistochemical staining of VEGF in rat kidneys (**d**). One representative of three independent experiments is shown. The result of Western blotting was quantified by densitometry. Data represent mean ± SE

We determined the abundance of nNOS and HIF-1α protein, markers for oxygen consumption and hypoxia, in the medulla and cortex of rat kidneys. As shown in Fig. 4, the expression of nNOS was down-regulated in both renal cortex and medulla in the 5/6 (A/I) model group as compared to that in the sham group. MLB up-regulated the expression of nNOS in renal cortex and medulla in 5/6 (A/I) operated kidneys (Fig. 4a and c). The expression of HIF-1α and VEGF was remarkably increased in renal medulla and only mildly increased in renal cortex in the 5/6 (A/I) model group as compared to that in the sham group (Fig. 4b). MLB only significantly reduced the expression of HIF-1α and VEGF in renal medulla but not in renal cortex in 5/6 (A/I) operated kidneys (Fig. 4b). Moreover, immunohistochemistry analysis showed that VEGF was stained in the tubule of sham-operated rat kidney, which became stronger in the CRF rat kidney and MLB attenuated VEGF expression in CRF kidney tissues (Fig. 4d).

## Discussion

In the current study, we showed that MLB improved renal function and attenuated renal fibrosis and inflammation in the 5/6 (A/I) rat model of CRF, which was correlated with the increase in renal blood flow and reduction in remnant renal oxygen consumption (QO_2_/T_Na_). Moreover, MLB reversed the expression of nNOS, HIF-1α and VEGF protein in the kidney of 5/6 (A/I) rats.

The kidney is one of the major organs with well-supplied oxygenated blood, and it is vulnerable to ischemic insults leading to impaired renal function [3].The ischemic conditions can be induced by acute kidney injuries and by damages associated with chronic kidney diseases [13]. Hypoxia, resulting from decreased blood flow or increased oxygen consumption, affects the expression of a wide array of genes, including many fibrogenic factors, leading to the progressive loss of renal function [4, 14, 15].MLB exerts beneficial effects in several rodent models of chronic kidney disease, and the protective mechanism of MLB in the kidney is related to its anti-oxidative and anti-fibrotic potentials [7–9]. It has been shown that MLB promoted renal circulatory state in healthy rat kidneys and adenine induced CKD kidneys [11, 15]. We therefore hypothesized that MLB protects renal function in chronic kidney disease through improving renal hemodynamics and subsequently attenuating hypoxia in 5/6 kidneys. In our study, we firstly showed that MLB attenuated renal function decline, renal fibrosis and inflammation in the 5/6 (A/I) rat model of renal failure. Secondly, we showed that MLB significantly increased renal blood flow. To further confirm that the renal hypoxia was reduced by MLB, we measured the expression of HIF-1α, the hypoxia marker, in the kidney tissues [5]. We found that MLB reduced the expression of HIF-1αin 5/6 rat kidneys suggesting that renal hypoxia was attenuated by MLB. Moreover, we measured the expression of VEGF which is a downstream target of HIF-1α in 5/6 and the renal expression of VEGF is tightly regulated by hypoxia [16] Our study showed that the renal expression of VEGF in CRF rats was reduced by the MLB treatment. Interestingly, in the present study, we found that MLB reduced renal oxygen consumption in 5/6 (A/I) rats. We further measure the expression of nNOS, a negative regulator of oxygen utilization in mitochondria, and we found that MLB restored the expression of nNOS in 5/6 (A/I) rat kidneys [4].

## Conclusions

In conclusion, MLB is renal protective and attenuates renal hypoxia in 5/6 (A/I) rats, and the mechanism is probably caused by improving renal hemodynamics and attenuating renal oxygen consumption.

## Abbreviations

5/6 (A/I): 5/6th renal Ablation/Infarction
A/I: Ablation/Infarction
BUN: Blood urine nitrogen
CKD: Chronic kidney disease
Col-I: Collagen-I
CRF: Chronic renal failure
CTGF: Connective Tissue Growth Factor
DBP: Diastolic Blood Pressure
FN: Fibronectin
FNa: Sodium filtered
GFR: Glomerular filtration rate
HIF-1α: Hypoxia inducible factor-1
IL-6: Interleukin-6
Male Sprague-Dawley rats: SD rats
MDA: Malondialdehyde
MLB: Magnesium Lithospermate B
nNOS: Neuronal nitric oxide synthase
O_2_ct: O_2_ content
QO_2_: O_2_ consumption
RBF: Renal blood flow
SBP: Systolic Blood Pressure
Scr: Serum creatinine
tHb: total arterial blood hemoglobin
UNaV: Sodium excreted in the urine
VEGF: Vascular Endothelial Growth Factor
